# Advances in Catheter Ablation: Atrial Fibrillation Ablation in Patients With Mitral Mechanical Prosthetic Valve

**DOI:** 10.2174/157340312803760767

**Published:** 2012-11

**Authors:** Pasquale Santangeli, Luigi DI Biase, Rong Bai, Rodney Horton, J David Burkhardt, Javier Sanchez, Justin Price, Andrea Natale

**Affiliations:** Texas Cardiac Arrhythmia Institute - St. David’s Medical Center, Austin (TX), USA

**Keywords:** Atrial fibrillation, mitral valve replacement, radiofrequency catheter ablation.

## Abstract

Atrial fibrillation (AF) is common in patients with mitral valve replacement (MVR). Treatment of AF in these subjects is challenging, as the arrhythmia is often refractory to antiarrhythmic drug therapy. Radiofrequency catheter ablation (RFCA) is usually avoided or delayed in patients with MVR due to the higher perceived risks and difficulty of left atrial catheter manipulation in the presence of a mechanical valve. Over the last few years, several investigators have reported the feasibility and safety of RFCA of AF in patients with MVR. Five case-control studies have evaluated the feasibility and safety of RFCA of AF or perimitral flutter (PMFL) in patients with MVR. Overall, a total of 178 patients with MVR have been included (21 undergoing ablation of only PMFL), and have been compared with a matched control group of 285 patients. Total procedural duration (weigthed mean difference [WMD] = +24.5 min, 95% confidence interval [CI] +10.2 min to +38.8 min, *P* = 0.001), and fluoroscopy time (WMD = +13.5 min, 95% CI +3.7 min to +23.4 min, *P* = 0.007) were longer in the MVR group. After a mean follow-up of 11.5 ± 8.6 months, 64 (36%) patients in the MVR group experienced recurrence of AF/PMFL, as compared to 73 (26%) patients in the control group, accounting for a trend toward an increased rate of recurrences in patients with MVR (odds ratio [OR] = 1.66, 95% CI 0.99 to 2.78, *P* = 0.053). Periprocedural complications occurred in 10 (5.6%) patients in the MVR group, and in 8 (2.8%) patients in the control group (OR = 2.01, 95% CI 0.56 to 7.15, *P* = 0.28). In conclusion, a quantitative analysis of the available evidence supports a trend toward a worse arrhythmia-free survival and a higher absolute rate of periprocedural complications in patients with MVR undergoing RFCA of AF or PMFL, as compared to a matched control group without mitral valve disease. These data would encourage the adoption of RFCA of AF in MVR patients mostly by more experienced Institutions.

## INTRODUCTION

Mitral valve disease is tightly associated with increased risk of atrial fibrillation (AF) [1], and open heart surgery for mitral valve replacement (MVR) conveys an additional risk of developing AF [1-3]. In these patients, AF has been also shown to be associated with poorer survival [3, 4]. Therefore, restoration and maintenance of sinus rhythm clearly represents the main therapeutic goal of AF treatment in patients with MVR, albeit pharmacologic rhythm-control strategies have been demonstrated largely ineffective to achieve lasting sinus rhythm maintenance [5]. Radiofrequency catheter ablation (RFCA) has been consistently demonstrated superior to antiarrhythmic drug therapy (AADs) for the long term maintenance of sinus rhythm in patients with AF [6], although the need for left atrial access and catheter manipulation in the presence of a prosthetic mitral valve has long been perceived as a relative contraindication to RFCA in patients with MVR. The risk of prosthetic valve dysfunction due to trauma from ablation catheter [7], and entrapment of the circular mapping catheter in the mitral valve apparatus [8] represent major concerns when performing RFCA of AF in MVR patients.

In recent years, several authors have reported the feasibility and safety of RFCA of AF in patients with MVR [9-12]. In the present article we will provide a state-of-the-art overview on the benefits and risks of RFCA of AF in patients with MVR, through a systematic revision of the available evidence.

## RADIOFREQUENCY CATHETER ABLATION IN PATIENTS WITH MVR: PUBLISHED EVIDENCE

To date, 4 multicenter observational series on RFCA of either AF [9-11] or perimitral flutter (PMFL)[12] in patients with MVR have been reported. All these series have been controlled with a matched group of patients without mitral valve disease and undergoing RFCA of AF in the same enrolling Institutions during the same study period (Table **1**). Lang *et al*. reported the first experience with RFCA of AF in 26 patients with mitral valve prostheses, comparing the acute and long-term procedural outcomes in these patients with those of 52 matched control patients with native mitral valves [11]. Warfarin was discontinued three days before the procedure and bridging with low-molecular-weight heparin was instituted in all patients. All patients underwent circumferential pulmonary vein ablation (CPVA) with a 8-mm solid-tipped ablation catheter, without confirmation of pulmonary vein isolation with a circular mapping catheter. Additional lines in the mitral isthmus and/or in the left atrial roof/floor were placed in the majority (81%) of patients. Patients with MVR had longer procedural (134 ± 25 min vs. 125 ± 31 min, *P* = 0.24) and fluoroscopy (35 ± 21 min vs. 21 ± 15 min, *P* < 0.001) times. After a mean follow-up of 10 months, AF recurred in 7/26 (27%) patients in the MVR group, as compared to 13/52 (25%) controls (Log-rank *P* = 0.658). Overall, periprocedural complications occurred in 3 (11.5%) patients in the MVR group, and consisted of a transient ischemic attack, of a femoral pseudoaneurysm, and of a failed transseptal access with aborted procedure.

In a subsequent multicenter observational controlled study, Lakkireddy *et al*. evaluated the benefits and risks of RFCA of AF in 50 patients with either a prosthetic mitral (82% of cases) or aortic valve [10]. All patients underwent pulmonary vein antrum isolation (PVAI) guided by recordings from a circular mapping catheter with intracardiac echocardiography (ICE) monitoring [6]. Patients with paroxysmal AF (40% of cases) underwent PVAI only, whereas in those with non-paroxysmal AF ablation was extended to the entire left atrial posterior wall down to the coronary sinus with adjuvant fractionated electrograms (CFAE) ablation.[6, 10] At the end of the procedure, all patients underwent challenge with high doses (up to 20 mcg/min for 15-20 min) of isoproterenol to disclose early pulmonary vein reconnection or non-pulmonary triggers, which were mapped and ablated. At variance with the series by Lang *et al*. [11], all patients in this study maintained periprocedural therapeutic warfarin [10, 13]. Total procedural (199 ± 49 min vs. 167 ± 28 min, *P* < 0.01) and fluoroscopy (60 ± 17 min vs. 54 ± 7 min, *P* < 0.01) times were confirmed longer in the MVR group, although no difference in arrhythmia-free survival was found between the two groups at 6-month follow up (22% vs. 16%, *P* = 0.60). Overall, complications occurred in 4 (8%) in the MVR group (2 arterio-venous fistulae, 1 cardiac tamponade requiring pericardiocentesis, and 1 phrenic nerve palsy) and in 2 (4%) control cases (1 pericardial effusion requiring pericardiocentesis, and 1 large groin hematoma requiring surgical evacuation and transfusion). Notably, no periprocedural thromboembolic event occurred in both groups. This finding highlights the thromboembolic protection associated with maintenance of periprocedural therapeutic warfarin, which has been consistently reported in multiple observational studies [13]. In the specific setting of patients with MVR, lack of warfarin discontinuation also minimizes the risk of prosthetic valve thrombosis.

Hussein *et al*. have recently confirmed such results in a series of 81 MVR patients [9] undergoing RFCA of AF with a protocol comparable to that of Lakkireddy *et al*. [10]. The control group in the study by Hussein *et al*. consisted of 162 age- and sex-matched controls without mitral valve disease. Overall, patients with MVR presented more commonly with concomitant atrial flutter (43.2% vs. 14.8%, *P* < 0.001), had lower left ventricular ejection fraction (49.2 ± 10.6% vs. 54.5 ± 8.5%, *P* < 0.001) and larger left atria (30.3 ± 8 cm2 vs. 23.1 ± 5.4 cm2, *P* < 0.001). Over a 24-month follow-up, patients with MVR had a higher arrhythmia recurrence rate compared to patients with native mitral valves (49.4% vs. 27.7%, *P* < 0.001). Procedure-related complications occurred in 3 (3.7%) patients in the MVR group and in 6 (3.7%) controls. Also in this study, no periprocedural stroke or transient ischemic attack was registered, further confirming the benefit of RFCA under therapeutic warfarin [13].

Mountantonakis *et al*. evaluated the feasibility, safety, and outcomes of PMFL ablation in patients with a history of mitral valve surgery (9 MVR and 12 repair with annuloplasty ring) [12]. In this study, an age-, gender-, and ejection fraction-matched control group of 21 patients without prior mitral valve surgery was included for comparison. No periprocedural complications occurred in both groups and, at a mean follow-up of 7 ± 4 months, no difference in arrhythmia recurrence rate was observed (29% in patients with prior mitral valve surgery vs. 33% in control patients, *P* > 0.99).

## RADIOFREQUENCY CATHETER ABLATION IN PATIENTS WITH MVR: SUMMARY OF THE EVIDENCE

As mentioned, the sample size of clinical studies on RFCA of AF in MVR patients was generally inadequate, with consequent lack of power to detect any real difference in treatment. A common approach to increase the sample size in order to increase the power to disclose clinically worthwhile differences is performing pooled analyses [13]. A dramatic example comes from a pooled analysis of clinical trials, published in the mid eighties, which evaluated thrombolytic therapy for acute myocardial infarction [14]. In this meta-analysis, Yusuf and colleagues reported a highly significant reduction in mortality with thrombolytic therapy, although only five of the 24 included trials had shown a statistically significant effect. The lack of statistical significance of most of the individual trials led to a long delay before the true value of thrombolysis was appreciated.

As specified, none of the studies on RFCA of AF in MVR patients reported significant differences in procedural outcomes when compared to matched control patients without mitral valve disease.

To further appraise the benefits and risks of RFCA of AF in patients with MVR, we performed a pooled analysis of procedural outcomes comparing patients with MVR with matched controls without mitral valve disease.

## DATA ANALYSIS

Data are expressed as odds ratio (OR) with 95% confidence interval (CI) for binary outcomes, and as weighted mean difference (WMD) and 95% CI for continuous outcome variables. Binary outcomes (i.e., AF recurrences and periprocedural complications) from individual studies were analyzed according to the Mantel-Haenszel model to compute individual ORs with pertinent 95% CI, and pooled summary effect estimate was calculated by means of a random-effect model, as reported [15, 16]. Weighted mean differences with pertinent 95% CI were computed for continuous outcome variables (i.e., total procedural and fluoroscopy times) by entering the mean and standard deviation of differences between baseline and follow-up, and were combined with a DerSimonian and Laird random effect method to obtain the summary estimate of the end-point, as reported [16]. Statistical level of significance was defined at a *P* < 0.05 [two tailed]. Analyses were performed using the STATA 11.2 software package (Stata Corporation, College Station, Texas, USA).

## PROCEDURAL DATA

**Fig. (1):**
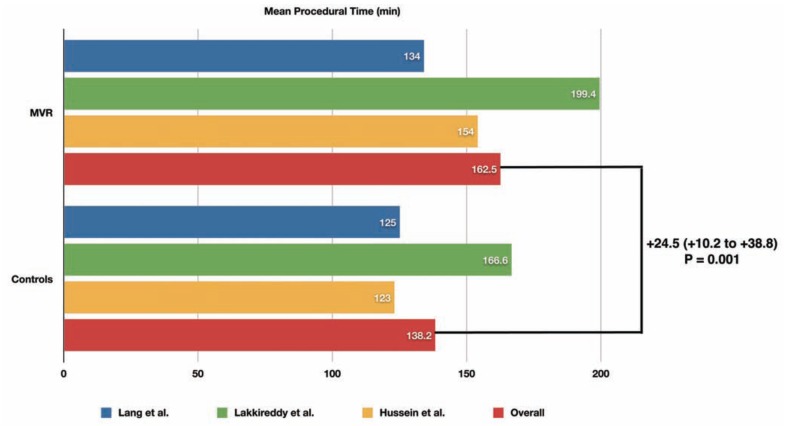
Mean total procedural time during RFCA of AF in patients with MVR compared to matched controls in each included study, and results of the pooled analysis. The weighted mean difference (WMD) and its 95% confidence interval is reported in bold.

**Fig. (2):**
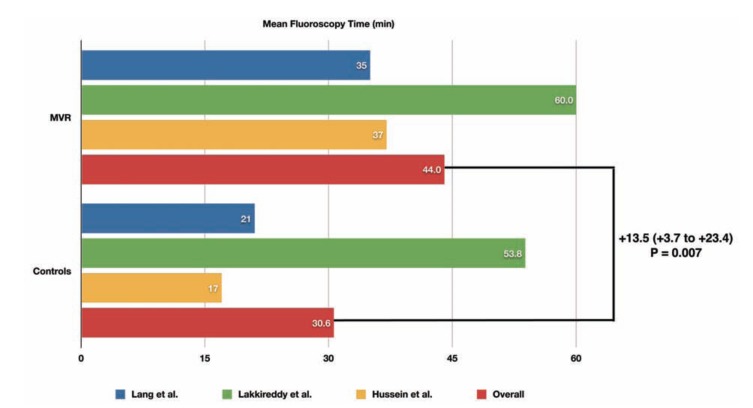
Mean total fluoroscopy time during RFCA of AF in patients with MVR compared to matched controls in each included study, and results of the pooled analysis. The weighted mean difference (WMD) and its 95% confidence interval is reported in bold.

A total of 463 patients undergoing RFCA of AF have been included in the pooled analysis, of whom 178 (38%) had MVR and 285 (62%) had native mitral valves. Compared to patients with native mitral valves, patients with MVR had longer total procedural (+24.5 min, 95% confidence interval [CI] +10.2 min to +38.8 min, *P* = 0.001) and fluoroscopy times (WMD = +13.5 min, 95% CI +3.7 min to +23.4 min, *P* = 0.007) (Figs. **1** and **2**).

## PROCEDURAL SUCCESS

**Fig. (3):**
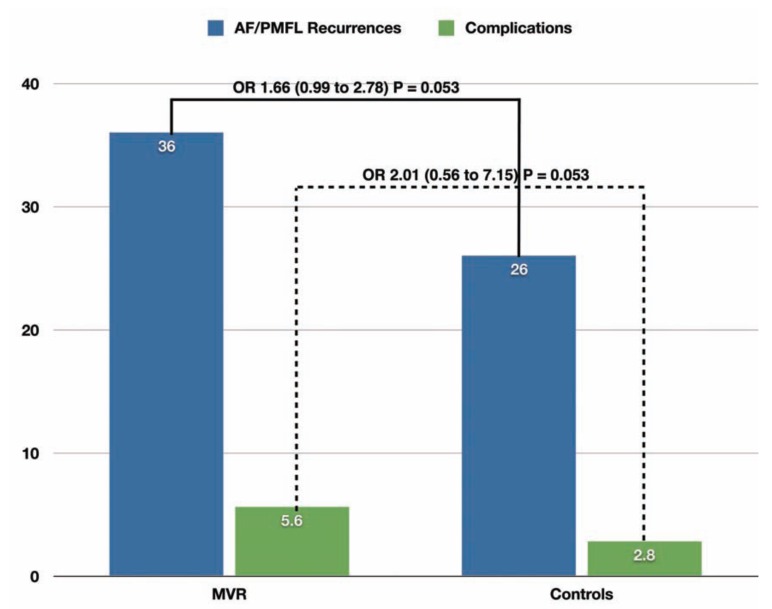
Pooled rate of atrial fibrillation (AF) / perimitral flutter (PMFL) recurrence and rate of total periprocedural complications in patients with MVR compared to matched controls. The pooled odds ratios and their 95% confidence intervals (MVR vs. controls) are reported in bold.

After an average follow-up of 11.5 ± 8.6 months, AF/PMFL recurred in 64/178 (36%) MVR patients, as compared to 73/285 (26%) patients with native mitral valves. These figures accounted for a trend toward an increased risk of recurrent arrhythmia after RFCA in MVR patients (OR = 1.66 [95% CI 0.99 to 2.78], *P* = 0.053) (Fig. **3**).

## COMPLICATIONS

Total periprocedural complications occurred in occurred in 10/178 (5.6%) patients in the MVR group, compared to 8 (2.8%) patients in the control group. Such numerically higher rate of complications in MVR patients did not reach the statistical significance at pooled analysis (OR = 2.01 [95% CI 0.56 to 7.15], *P* = 0.28) (Fig. **3**).

## COMMENT

Atrial fibrillation is the most common arrhythmia following surgery for valvular heart disease [17], with a cumulative risk of new-onset arrhythmia reaching 23% over a span of 5 to 10 years [18]. In patients with MVR, the presence of AF is associated with a strikingly higher risk of morbidity and mortality [19, 20]. With these premises, restoration and maintenance of sinus rhythm represent the main therapeutic goal in patients with MVR. RFCA has been demonstrated the most effective treatment to restore and maintain sinus rhythm in different clinical settings, although its benefits in patients with MVR have not been adequately investigated. Recently, few high-volume and highly experienced Institutions have reported the results of RFCA of AF/PMFL in patients with MVR [9-12]. Although none of such studies reported significant differences in outcomes as compared to matched control patients with native mitral valves, the sample size was largely inadequate to draw definite conclusions. Notably, at pooled analysis, both AF/PMFL recurrences and periprocedural complication rates were numerically higher in patients with MVR compared to matched controls with native mitral valves.

In fact, there was a trend toward a significantly increased risk of recurrences in patients with MVR (OR = 1.66 [95% CI 0.99 to 2.78], *P* = 0.053). In this regard, it is important to point out that the majority of the studies adopted pulmonary vein isolation as the main approach to RFCA in these patients. Whether the procedural success might improve adopting more extensive ablation approaches (e.g., posterior wall isolation, more aggressive non-pulmonary vein trigger ablation) warrant further investigations.

With regard to complications, patients with MVR had a double rate of total periprocedural complications when compared to control patients (i.e., 5.6% vs. 2.8%, *P* = 0.28). Despite the inclusion of more than 460 patients, even our present analysis might be underpowered to disclose actual differences in outcomes, and lack of statistically significant differences in complications rates should not be viewed as lack of clinically relevant differences. As mentioned, such results were obtained in highly experienced Institutions, and the extent to which they could be generalized to other less experienced operators or Institutions warrants further investigations.

## CONCLUSIONS

Catheter ablation of AF in patients with MVR is feasible and relatively safe in experienced Institutions, although both post-ablation arrhythmia recurrences and periprocedural complications tends to be higher compared to matched controls with native mitral valves. These data would support the widespread adoption of RFCA of AF in patients with MVR mostly by experienced operators or Institutions.

## Figures and Tables

**Table 1.** Characteristics of Studies on Radiofrequency Catheter Ablation of Atrial Fibrillation in Patients with Mitral Valve Replacement

Study Ref. #	Year	Design	N. pts (MVR)	N. pts (CTRL)	Abl. technique	FU (months)
Lang *et al*. [11]	2005	Case/Ctrl	26	52	CPVA + MIL (81%) + PL	10
Lakkireddy *et al*. [10]	2011	Case/Ctrl	50	50	PVAI + CFAE (NPAF) + Non-PV Triggers	12
Hussein *et al*. [9]	2011	Case/Ctrl	81	162	PVAI	24
Mountantonakis *et al*. [12]	2011	Case/Ctrl	21	21	MIL	6

N. = number; pts = patients; MVR = mitral valve replacement; CTRL = controls; Abl. = ablation; FU = follow-up; CPVA = circumferential pulmonary vein ablation; MIL = mitral isthmus line; PL = posterior left atrial line; PVAI = pulmonary vein antral isolation; CFAE = complex fractionated atrial electrograms.
